# In the Field Feasibility of a Simple Method to Check for Radioactivity in Commodities and in the Environment

**DOI:** 10.1371/currents.dis.07059b54a787dcfcf53ac46ab5a6a809

**Published:** 2017-05-30

**Authors:** Stefano Alessandri

**Affiliations:** Department of Statistics, Computer Science, Applications "Giuseppe Parenti", University of Florence, Florence, Italy

## Abstract

**Introduction:**

Some release of radionuclides into the environment can be expected from the growing number of nuclear plants, either in or out of service. The citizen and the big organization could be both interested in simple and innovative methods for checking the radiological safety of their environment and of commodities, starting from foods.

**Methods:**

In this work three methods to detect radioactivity are briefly compared  focusing on the most recent, which converts a smartphone into a radiation counter.

**Results:**

The results of a simple sensitivity test are presented showing the measure of the activity of reference sources put at different distances from each sensor.

**Discussion:**

The three methods are discussed in terms of availability, technology, sensitivity, resolution and usefulness. The reported results can be usefully transferred into a radiological emergency scenario and they also offer some interesting implication for our current everyday life, but show that the hardware of the tested smart-phone can detect only high levels of radioactivity. However the technology could be interesting to build a working detection and measurement chain which could start from a diffused and networked first screening before the final high resolution analysis.

## Introduction

The number of nuclear plants in the world, either in or out of service, is growing and many of them are built with obsolete technology and are becoming older and older 1. Therefore some release of radionuclides into the environment can be expected in the future.

For this reason, the average (informed) citizen and big organizations could both be interested in innovative and simple methods to check the radiological safety of the local environment and of commodities, starting from foods. Traditional methods require special expertise with expensive and heavy devices and for this reason they are not very popular and only specialized organizations can afford them. On the other hand, if detecting radioactivity were as simple and cheap as measuring atmospheric pressure or body temperature, it could raise a great interest especially in our networked society and particularly during a radiological emergency.

In recent years, an astounding availability of portable and wearable 2 sensors has flooded the consumer market, as an effect of the explosion of the smartphone market.

Almost anyone can currently monitor georeferenced environmental parameters and can find out and share their measurements in real time 3.

The parameters that can be more easily measured by a smartphone and the appropriate apps installed and running are: local magnetic field (Intensity and direction), visible light (intensity and composition in terms of three-component colorimetric measurement), sound (level and spectrum), atmospheric pressure, atmospheric humidity, latitude, longitude, speed, acceleration, local gravity. In the same way it is well known that almost everyone can take and share in real time georeferenced photos, videos and sound recordings.

It is less widely known that with the same hardware (a smartphone with a built-in camera) and the appropriate software, anyone can monitor radioactivity and measure its intensity, although with sensitivity limitations 4^,^^5^^,^^6^^,^^14^.

In this work, three methods for radioactivity detection and measurement in commodities and in the environment are briefly compared and discussed. They are different in technology, sensitivity, resolution, and cost.

A method that is very recent relies on the current smartphone technology, with no need for additional hardware, which makes it cheap and widely available. It could be valuable for a wide screening activity and for the production, spreading and sharing of information 7. The aim of this work is to verify its putative usefulness in the field.

The two other methods are well known in the specialized laboratory and depend on specialized instruments. They were adopted here as a reference to evaluate the usefulness of the method based on the smartphone technology.

## Methods

**The Reference Sources**

To test and calibrate the detectors adopted in this work, a reference standard, characterized by a weak and safe (NRC/IAEA/EU exempt quantity) but clearly detectable gamma ray emission, was needed. A set of eight factory-calibrated point sources was chosen: the RSS8UN set by Spectrum Techniques, LLC., including Ba-133 (t_1⁄2_ = 10 year), Cd-109 (t_1⁄2_ = 462.6 day), Co-57 (t_1⁄2_ = 271 day), Co-60 (t_1⁄2_ = 5.27 year), Mn-54 (t_1⁄2_ = 312 day), Na-22 (t_1⁄2_ = 2.6 year) and Zn-65 (t_1⁄2_ = 244 day) at 3.7×10^4^ Bq on May 2015, Cd-109 (t_1⁄2_ = 463 day) and Na-22 (t_1⁄2_ = 2.6 year) at 3.7×10^4^ Bq on Apr 2015 and Cs-137 (t_1⁄2_ = 30 year) at 3.7×10^3^ Bq on Apr 2015.

**The Instruments**

The first kind of detector adopted was based on smartphone technology.

Three common models were tested. They were equipped with a specialized app: "RadioactivityCounter" 4^,^^8^ (http://www.hotray-info.de/). The app is available for Android and iOS systems.

The adopted models were Samsung S4, Samsung S7 and Samsung A3.

The S4 is equipped with a 4.4x3.4=15 mm^2^ sensor (resolution 4128 x 3096 = 12780288 pixel), the S7 is equipped with a 4.2x3.1=13 mm^2^ sensor (resolution 4032 x 3024=12192768 pixel), the A3 is equipped with a 3.6x2.7=9.7 mm^2^ sensor (resolution 3264 x 2448 = 7990272 pixels).

CCD or CMOS chips, used as digital image sensors in surveillance or in smartphone cameras, are sensitive not only to visible light but also to higher energy photons. The software analyzes the signals produced by the front or by the rear camera of the smartphone, which has been previously shielded from visible light by an alluminium foil, subtracts the thermal noise and estimates the gamma-ray exposure of the sensor. Furthermore the background emission (measured before) can be subtracted.

The second kind of detector was the Geiger counter PRD 100 (http://www.prd100.com/), made by ITS srl. This device is equipped with a Geiger-Müller tube of 111 mm length x 11 mm diameter (max. section 1221 mm^2^), which is small enough to allow full portability but whose section is much larger (82 times) than the section of the largest smartphone sensor (15 mm^2^).

This detector shows additional interesting features: it is cheap (around 100 €), it is small and easily portable (190 g, 123 mm x 91 mm x 35 mm) it works with rechargeable standard batteries (3 AA), it can work in stand-alone mode and/or connected with a smartphone via a Bluetooth radio interface.

A dedicated app (Marie pro PRD-100) running on the smartphone provides some essential real time radiation counting and data displaying, saving, sharing and geo-referencing.

The third kind of instrument chosen in this work was a 1024 channel NaI(Tl) gamma spectrometer made by Ortec, priced around 18000 €.

The system hardware consists of a thallium-doped sodium iodide detector enclosed in a low-background lead shield (30 mm thick), an analog-to-digital converter (ORTEC DigiBase) integrated in an all-in-one spectrometer, and a laptop PC. The digiBASE supplies the multi-channel analyzer function, the high voltage for the NaI(Tl) detector, and all the signal processing electronics. The internal stabilization electronics and the internal check source (K-40 4500 Bq/kg) allow the system to be used over a wide range of environmental conditions. However it can be hardly defined portable, if the 80 kg lead shield is taken into consideration. The NaI(Tl) crystal is a 76.2 mm height x 76.2 mm diameter (3” x 3”) standard. The digiBase is connected to the control computer via a USB interface, which powers the whole system.

Several proprietary software components control the instrument, from the first setting to the final analysis. Ortec MCB Connections-32 acts as a first-level connection driver for the DigiBase. Maestro-32 MCA Emulation Software provides the second-level control of the DigiBase, the live spectral display and the automatic control of acquisition and analysis. This is achieved via a graphical user-programmable interface or via pre-programmed job streams. The software provides also data and results printing and storage. NuclideNavigator is an interactive gamma-ray reference and library program to view, query, and extract gamma-ray energies and yields, half-lives and parent/daughter relations from databases. It can be used to build application libraries or working libraries. ScintiVision-32 is an integrated multi-channel analyzer (MCA) emulator and gamma-spectrum analysis program. It integrates Maestro-32 functions and manages the collection and analysis of gamma-ray spectra. It includes commands that allow you to edit nuclide libraries and automated command sequences or “job streams.”

The set of technologies described regarding gamma-spectroscopy with sodium iodide scintillator allows the identification and quantitative determination of gamma ray emitting radioisotopes, either natural ones such as K-40, U-238, Th-232 or anthropic ones such as Cs-137 and I-131 9, single or in simple mixture 10.

A fourth method must be cited, though it was not experienced in this work, because it represents the state-of-the art in gamma-ray spectroscopy. It is based on High Purity Germanium (HPGe) detectors and can give excellent gamma signal resolution in the whole spectral window, even for energies as low as 3 keV, where NaI(Tl) detectors cannot usefully work and gives a resolution 16 times better than NaI(Tl). The major drawback of germanium detectors is that they must be cooled to liquid nitrogen temperature to produce spectroscopic data. An HPGe system is more complex to manage and on average is priced five to ten times as much as a NaI(Tl) system.

**The radon issue**

The Radon is a ubiquitous gas, mainly derived from the natural U-238 decay chain.

Radon isotopes emit alpha particles but some radionuclides from Radon's progeny (Pb-214, Bi-214) emit gamma rays and can add their signals to the spectrum of a sample. To address this problem an independent Radon sensor was adopted to estimate the radon concentration during the analyses by the NaI(Tl) system.

The sensor adopted is the Rstone by Rsens. It can be connected to a computer by a proprietary USB pen. A proprietary program can read and analyze the data stored in the sensor's memory. The sensor itself runs on a battery whose charge can guarantee up to two weeks of continuous autonomy and monitoring, and has a display to check the current Radon concentration measured during the last 30'.

**The Background management**

With the “RadioactivityCounter” app running on a smartphone, the sensor noise and the background emission can be measured and stored in the memory, and can be subtracted from each sample measure. Measures are expressed in CPM and saved as total and by-minute counts, together with the sensor temperature (that can affect the measure).

No background compensation is provided by the PRD-100 Geiger counter and by the corresponding Marie PRO PRD-100 app running on a smartphone connected to the instrument. The counting is only instantaneous.

The NaI(Tl) gamma spectroscopy systems allow full control of the background.

The instrument is protected by a lead shield that effectively prevents the variable environmental background radiation from hitting the sensor. The shield itself is a source of radiation mainly from U-238, and Th-232 decay series; anyway this radiation is constant (except for the environmental radon contribution) and can be measured and subtracted with high reliability and precision, acquiring “blank” spectra periodically. The K-40 internal standard can also be included in the background. Therefore a spectrum of the background and of the internal standard was acquired for 963933 s, checking for its stability (on the Energy axis) and saving the result every 1800 s. The stability was assessed by continuously keeping the laboratory temperature as close as possible to 295K and monitoring the stabilizer of the instrument, locked to the K-40 peak: if some adjustment occurred, then the corresponding 1800 s set of data were discarded. This way, the centroid of the photopeak of the K-40 at 1461 keV was always kept corresponding to the channel 552.20+/- 0.20.

**The Calibration of the NaI(Tl) system**

The spectrum described above, was used either for the background subtraction or for the first calibration step 11 and all the following spectra were acquired with the constraint of the K-40 centroid corresponding to channel 552.20 +/-0.20.

The energy calibration of the NaI(Tl) system was done in several steps. For a first energy calibration the K-40 peak (4518 Bq) was considered with some clearly recognizable peak of the background: the Tl-208 peak at 2614 keV, near the high energy end of the spectral window , from the Th-232 decay chain, which is widely used as a gamma tracer of natural thorium 12^,^^13^ and the Bi-214 peak at 1765 keV which is widely used as a gamma tracer of natural uranium 12^,^^13^.

During the second step the energy calibration was refined by considering, from the same background spectrum, the Ac-228 peak at 969 keV, the Tl-208 peak at 511 keV and the Pb-212 at 239 keV from the Th-232 decay chain.

For the final energy calibration steps, three reference sources were chosen and their corresponding spectra were acquired: Ba-133 (53 and 81 keV peaks), Cd-109 (88 keV peak) and Cs-137 (662 keV peak). The spectrum of each source was acquired separately, for 66691 s (Ba-133 and K-40), for 14189 s (Cd-109 and K-40) and for 81453 s (Cs-137 and K-40). The three point sources were acquired without any correction for the geometry, at 75mm from the detector surface, to lower the intensity of their signals and make it possible for the stabilizer to work properly.

The final calibration is reported below, where “Channel” is the integer index of the channel ranging from 0 to 1023:

Energy = -7.7883 +2.568091*Channel +0.000165948*Channel^2^

The full width at half maximum (FWHM) was calibrated as a linear function of energy:

FWHM = 4.8213 +0.038887*Channel

The Efficiency was factory-calibrated.

**The test of smartphone sensitivity**

The rear (main) camera of each smartphone was shielded from light using a piece of aluminium foil. The “Radiation Counter” app was loaded and launched and the shielding effectiveness was verified by assessing that the background remained unchanged also putting the shielded lens near a strong light lamp. The activity of the Na-22, Zn-65 and Cs-137 sources were measured putting them at 75, 30, 15, 5, 0 mm from the lens' surface of the rear camera of each smartphone for more than 1200 s.

The Background was checked by putting the radioactive sources at a distance greater than 4m.

The same protocol was followed with the Geiger counter, referring the distances listed above to the instrument surface that directly covered the Geiger-Müller tube.

## Results

In the laboratory where the NaI(Tl) spectrometer works and where the radiation counters were tested, the radon concentration varied between 20 and 60 Bq/m^3^ during the testing periods and at this level did not require any special attention. The main environmental problem was to keep the temperature stable to have the NaI(Tl) spectrometer stable as well.

Only the strongest radioactive reference source (Na-22) could induce a response that was clearly different from the background in all the counters, smartphones included, as reported in Fig. 1 and in Tab. 1. Figure 1 and Table 1 show graphically (Fig 1) and in tabular format (Tab. 1) the measures obtained from each counter, at different distances from the Na-22 reference source.

**Graph showing the relationship between counts and distance, for each counter, from the Na-22 reference source.**
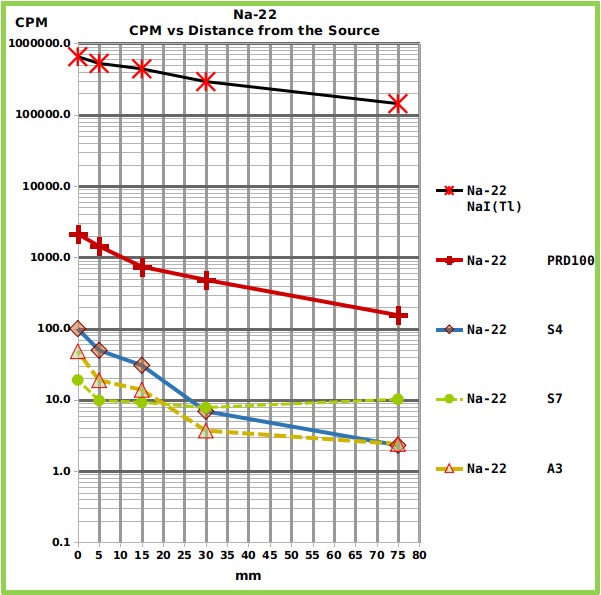
The Graph shows the Counts Per Minute (CPM) of the four tested radiation counters and of the NaI(Tl) spectrometer, put at different distances from Na-22 reference source of gamma rays. The numerical values are reported in Tab. 1.

**Figure:**
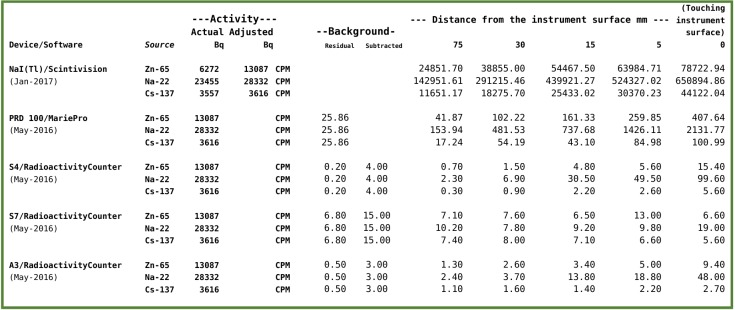
**Table 1:** The Table shows the output, expressed in CPM (Counts Per Minute) of the tested radiation counters and of the tested NaI(Tl) spectrometer, put at different distances from three different reference sources of gamma rays. For the NaI(Tl) spectrometer, the counts from the whole spectral windows were considered and the output adjusted for the half-life of the nuclides and for the different acquisition date.

The NaI(Tl) sensor showed a very different sensitivity.

For the sake of comparison, the photo-peak of the reference source of Cs-137 (3700 Bq), put on the surface of the NaI(Tl) sensor (18200 mm^2^) gave a count of 1.31*10^4^ CPM and was “viewed” by the spectrometer as a 1L sample containing 7262 Bq/kg of Cs-137 (Fig. 2). This kind of instrument easily recognizes contents as low as the Tl-208 of the background, “viewed” as a concentration of 19.4 Bq/kg and whose photo-peak area corresponds to 6.60*10^-2^ CPM (Fig. 2

**Gamma-ray spectrum of the Cs-137 reference source:**
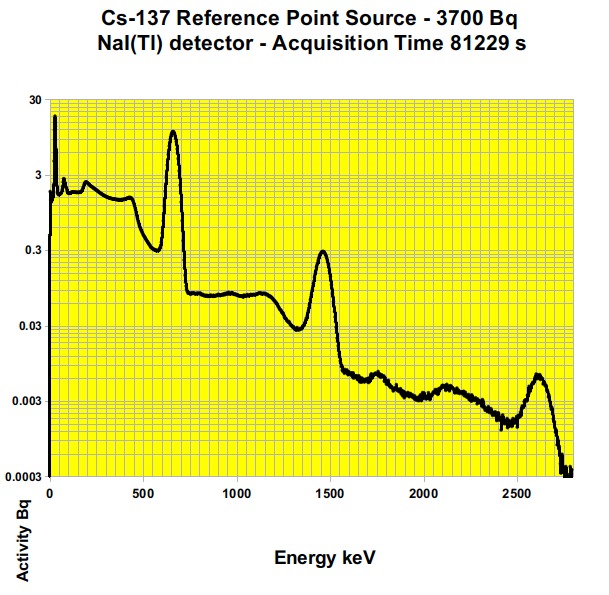
Output of the NaI(Tl) Gamma-ray spectrometer. The point source of Cs-137 (t1⁄2 = 30 year, 3.7×103 Bq on Apr 2015, photo-peak 662 keV) was placed on the sensor surface. Also the photo-peaks at 2614 keV (Tl-208 from the Lead shield) and at 1461 keV (K-40 internal standard) can be clearly observed.

The same reference source of Cs-137 (Fig 3 and Tab. 1) was not sensed by the S7 and was hardly sensed by the A3 and only within a distance equal or less than 5 mm. Only the S4 performed reliably.

**Graph showing the relationship between counts and distance, for each counter, from the Cs-137 reference source.**
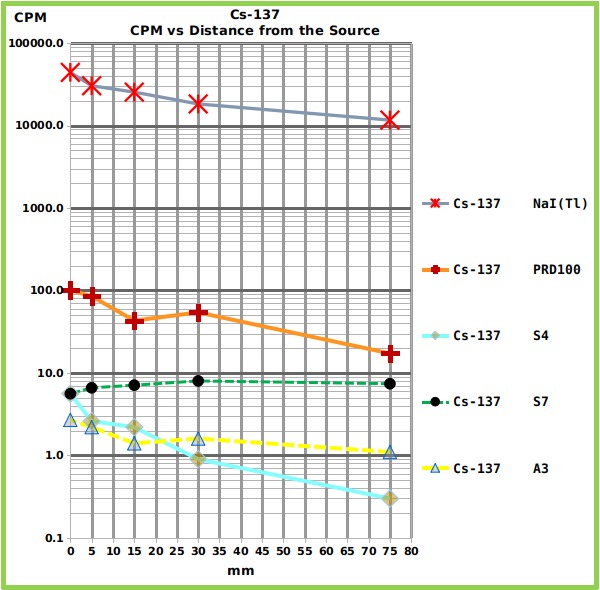
The Graph shows the Counts Per Minute (CPM) of the four tested radiation counters and of the NaI(Tl) spectrometer, placed at different distances from Cs-137 reference source of gamma rays. The numerical values are reported in Tab. 1.

The Zn-65 reference source (Fig. 4 and Tab. 1) showed intermediate counts between the strongest (Na-22) and the weakest source (Cs-137).

**Graph showing the relationship between counts and distance, for each counter, from the Zn-65 reference source.**
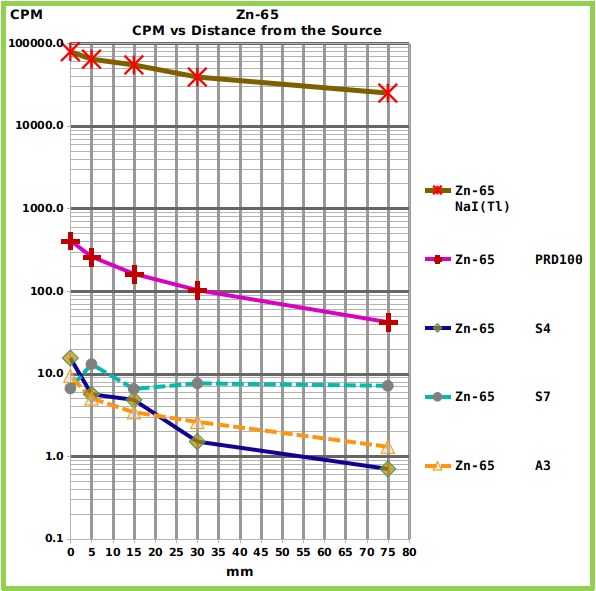
The Graph shows the Counts Per Minute (CPM) of the four tested radiation counters and of the NaI(Tl) spectrometer, placed at different distances from Zn-65 reference source of gamma rays. The numerical values are reported in Tab. 1.

The three smartphones performed very differently. The most recent one (Samsung S7), which is equipped with the most advanced (and small) sensor showed too much background thermal noise and was not able to sense the reference sources (Fig. 5 and Tab. 1) , except for the strongest (Na-22) at the minimum distance. On the other hand, only the S7, running under Android 6 (the A3 and the S4 runs under Android 5) showed no problem with file saving.

**Graph showing the response of the S7 smartphone to the different reference sources at the different distances.**
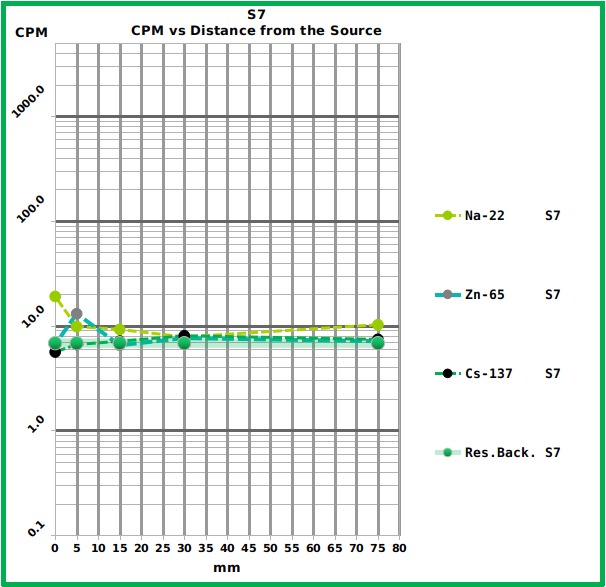
The Graph shows the Counts Per Minute (CPM) of the three tested radiation sources placed at different distances from the S7 surface. The numerical values are reported in Tab. 1. The residual background level is also reported.

Among the tested smartphones the most reliable was the S4 which showed a good sensitivity and whose counts followed the expected trend (Fig. 6 and Tab. 1). The A3 showed less sensitivity and performed worse (Fig. 7 and Tab. 1).

**Graph showing the response of the S4 smartphone to the different reference sources at the different distances.**
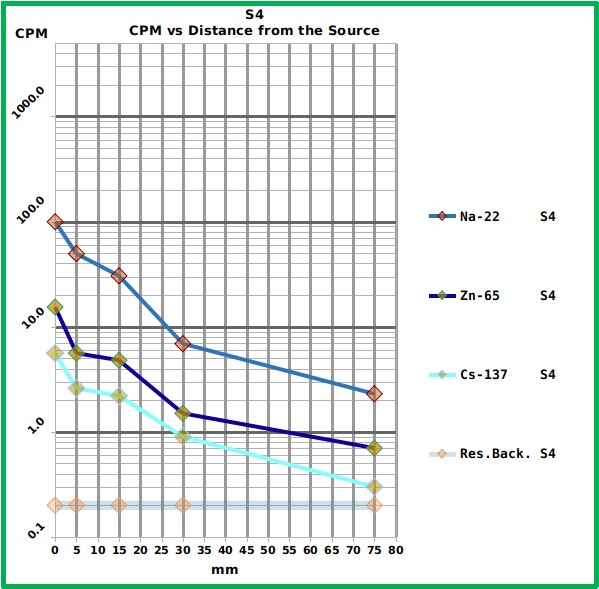
The Graph shows the Counts Per Minute (CPM) of the three tested radiation sources placed at different distances from the S4 surface. The numerical values are reported in Tab. 1. The residual background level is also reported.

**Graph showing the response of the A3 smartphone to the different reference sources at the different distances.**
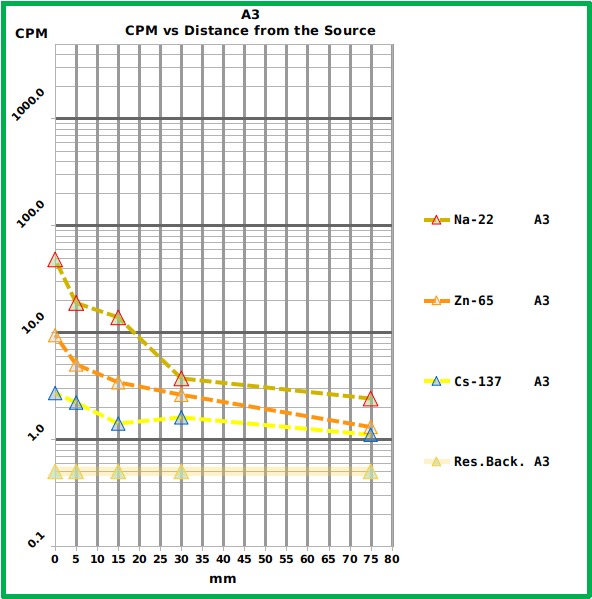
The Graph shows the Counts Per Minute (CPM) of the three tested radiation sources placed at different distances from the A3 surface. The numerical values are reported in Tab. 1. The residual background level is also reported.

The Geiger counter PRD100 (Fig. 8 and Tab. 1) showed a much higher (one order of magnitude) sensitivity of the best performing smartphone (S4) but the software running on the connected smartphone lacks some useful feature which is present in the "RadiationCounter" app. (that is to say data-saving in CSV standard format, a count every 60 s, temperature and battery status and, for each measuring session, total duration, background, noise related to the border of the sensor). Furthermore the app allows real-time background subtraction, 60 s to 1800 s means and general mean calculation.

**Graph showing the response of the PRD100 Geiger counter to the different reference sources at the different distances.**
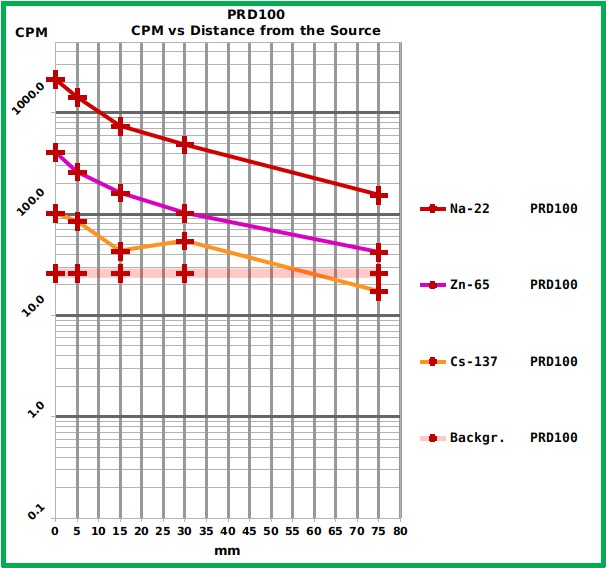
The Graph shows the Counts Per Minute (CPM) of the three tested radiation sources placed at different distances from the PRD100 surface. The numerical values are reported in Tab. 1. The residual background level is also reported.

The NaI(Tl) spectrometer showed (as expected) the best sensitivity (Fig. 9 and Tab. 1) and the highest reliability with the best background management.

**Graph showing the response of the NaI(Tl) spectrometer to the different reference sources at the different distances.**
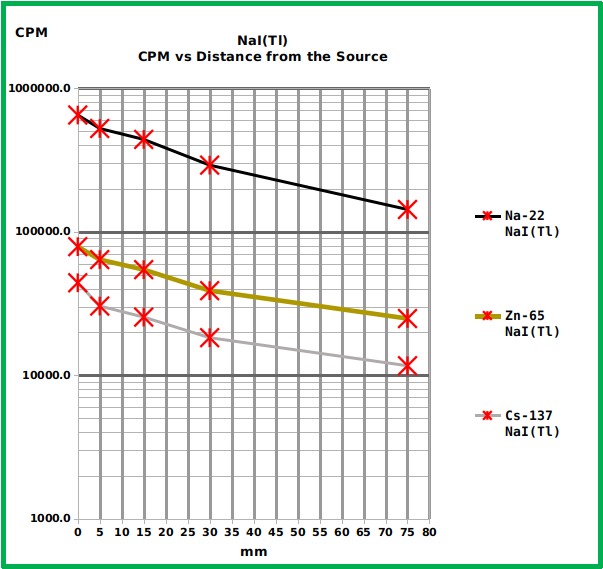
The Graph shows the Counts Per Minute (CPM) of the three tested radiation sources put at different distances from the surface of the sensor of the NaI(Tl) gamma spectrometer. The total counts from the whole spectral window are considered to allow a proper comparison with the other devices. The numerical values are reported in Tab. 1. The background is not reported here because it depends on the K-40 internal standard and on the lead shield, not on the external environment and it can be evaluated observing the spectrum reported in Fig. 2.

## Discussion

The current smartphone technology could open up to a massive radiation monitoring, with some limitations due to low sensitivity, where the number of data could compensate for the possible lack of precision and puts in the hands of the average citizen a direct knowledge that previously only specialized entities could access.

Furthermore, the fact that anyone can check for the safety of the nearby environment leads to a new kind of bottom-up control of the information released by interested stake-holders and by the public authorities, allowing more transparent decision making.

According to the United Nations Scientific Committee on the Effects of Atomic Radiation (UNSCEAR) the worldwide average natural dose to humans is about 2.4 mSv/y (millisievert per year) 15.

On the other hand, the International Commission on Radiological Protection (ICRP) recommends effective dose limits to reduce the risk of stochastic effects to tolerable levels 16 for members of the general public in planned exposure situations and proposes as first reference level 1 mSv/y. The Commission considers also existing exposure situations that are defined as "those that already exist when a decision on control has to be taken" and explains that "there are many types of existing exposure situations that may cause exposures high enough to warrant radiological protective actions, or at least their consideration". The Commission proposes the reference interval between 1 and 20 mSv/y for these conditions that can be due to NORM (Naturally Occurring Radioactive Material), natural background radiation and radioactive residues within the human habitat. The Commission defines also the levels between 20 mSv/y to 100 mSv/y as "reference levels for the highest planned residual doses in emergency situations". Therefore according to UNSCEAR and ICRP, an annual dose ten times the "worldwide average natural dose to humans" is already an emergency.

According to our data, at least one of the tested smartphone (S4) is able to reliably distinguish between a residual background of 0.2 CPM and a tenfold greater gamma emission (Tab. 1 and Fig. 6). The cited background can be assumed to be near the average value, according to UNSCEAR's definition and according to the information available on the geographic area where the tests were done: the alluvial plain of Florence-Prato-Pistoia 17^,^^18^. Therefore, in such conditions, the device could be used to detect an environmental radiation level that could signal a radiological emergency, according to ICRP's definition 16. On the other hand, no real-time detection can be expected in low exposure conditions because to achieve the reported results, several minutes were needed for each measure and all the measured counts, except the S4 measure of the Na-22 put directly on the smartphone surface (99.6 CPM) are less than one count per second. If we consider that one count per second is equal to 60 CPM that in turn, in this case, corresponds to 300 times the residual background, then it can be concluded that only high levels of gamma activity can be detected in real-time by this kind of device and that the low sensitivity of the small sensor of a smartphone can be a serious drawback, that could be compensated only by the adoption of an external sensor. The low sensitivity would not be a problem in a severe radiological emergency, where the usefulness of many working detectors should be unquestionable and where almost everybody could become an active node in an environmental sensory network. In this scenario the value of the information collected can only be imagined.

Of course the huge amount of environmental data that could be produced and shared needs some public revision and lab validation, and here a sensitive, traditional and inexpensive technology like the NaI(Tl) spectrometry can have a new role, before the definite Hi-Res confirmation that only the HPGe technology can give.

## Corresponding Author

Stefano Alessandri, Department of Statistics, Computer Science, Applications "Giuseppe Parenti", University of Florence, Florence, Italy. Email: stefano.alessandri@unifi.it

## Data Availability

The data are freely available in figshare repository and can be accessed via the following links:

- Summary of smartphones, geiger counter and NaI(Tl) spectrometer dataset: https://doi.org/10.6084/m9.figshare.4555507.v1

- NaI(Tl) raw dataset: https://doi.org/10.6084/m9.figshare.4644997.v1

## Competing Interests

The author has declared that no competing interests exist.
